# The impact of public policy of family doctor contracting on medical expenses from the perspective of residents at community level in China

**DOI:** 10.3389/fpsyg.2022.972904

**Published:** 2023-01-04

**Authors:** Lele Li, Xiaotong He, Caihua Zhang

**Affiliations:** ^1^School of Labor and Human Resources, Renmin University of China, Beijing, China; ^2^School of Politics and Public Administration, Southwest University of Political Science and Law, Chongqing, China

**Keywords:** family doctor contract, community level hospital, medical resource allocation, chronic diseases, residents

## Abstract

**Objective:**

To analyze the impact of the contract of family doctors in community-level in Beijing on residents’ medical expenses, so as to explore the efficacy of family physician contract service to facilitate the hierarchical treatment system. This study pioneeringly used the panel data of medical records and medical insurance settlement details provided by a community hospital in Beijing. By analyzing the correlation between family doctors and medical expenses, the study provided empirical support for the advancement of family doctor contracting in China.

**Methods:**

Using the panel data of medical records and medical insurance settlement details of 5,851 residents in a community hospital in Beijing from January 2018 to May 2021 for a total of 41 months, mixed-effects model and random-effects model were used for analysis.

**Results:**

The contract of family doctor has significantly increased residents’ medical expenses. The types of medical insurance and whether patients with chronic diseases have a significant impact on residents’ medical expenses.

**Conclusion:**

Family doctor contracting increases the probability of residents seeing a doctor in community hospitals. Community family doctor contracting services should be vigorously promoted, especially for special groups such as chronic disease patients and the elderly. The increase in the contracting rate of family doctors will become an important starting point for promoting hierarchical medical system and increasing the primary treatment at the community-level.

## Introduction

The contract of family doctor is an effective way to improve hierarchical treatment system, and integrate and optimize the allocation of medical resources, so as to improve the accessibility of medical services to the residents at grassroot. It has played an important role in the medical and health systems of many countries in the world (Yuan et al., 2020). International experience has shown that family doctors, as “health gatekeepers,” can play a key role in hierarchical treatment (Macinko et al., 2003; Velasco et al., 2011; Pedersen et al., 2012). The family doctor system can promote the two-way referral between community health service institutions and the hospitals of three different grades, enabling community residents to resolve minor illnesses nearby and turn serious illnesses to specialized hospitals (Huang et al., 2019). At present, the contract of family doctors in China includes basic medical services and basic public health services. In addition to these basic services, personalized contract services are also provided. Personalized contract service is to formulate targeted service according to the differentiated health needs of residents. At the same time, the government has delegated its basic public service functions to community health service agencies, and paid close attention to the signing rate to ensure the smooth implementation of the family doctor contract.

At present, the policy has paid more attention to the contract rate of family doctors. Domestic scholars have studied the relationship between the contract rate of family doctors and residents’ visits to community hospitals and residents’ needs for related services. However, the existing research mainly analyzes the influence of community family doctor contracting on the residents’ willingness to seek medical treatment in community health service institutions through questionnaires, selection experiments, etc. But there is a lack of analysis on the actual medical treatment behavior of residents based on the actual data of medical treatment and medical insurance settlement. Therefore, the main research questions of this paper include: First, what is the impact and the impact mechanism of family doctor contracting on residents’ medical expenses? Second, what is the impact and impact mechanism of contracting family doctor services on the medical expenses of the elderly and chronically ill patients?

Although there are many domestic articles published in past few years, those articles basically depended on case studies, questionnaire surveys, and so on. The above data collected in the form of patient self-report may have some bias. Also, ethical issues such as patient privacy protection policies further exacerbate the difficulty of data collection. Whether family doctors are helpful to advance hierarchical treatment mechanism in China is still lack of empirical evidence. Besides, the impact of family doctors among different groups also varies. What kinds of factors interfere with essential groups’ medical expenses still confusing and needs to be tested empirically based on actual medical record or insurance settlement data.

This study uses the actual medical treatment data and medical insurance settlement data of residents in a community hospital in Beijing. By matching the two kinds of data, this study empirically analyzes the impact and mechanism of family doctor contracting on residents’ medical expenses. Also, it provides evidence support for improving the coverage of primary treatment in community hospitals and promoting the hierarchical treatment system.

## Literature review

The signing of family doctor solidifies the contractual relationship between community residents and general practitioners through the form of contract, which can effectively improve the continuity of patient nursing (Zhao et al., 2015). While the continuity of care is considered as a cornerstone or core value of primary care (Saultz and Albedaiwi, 2004; Gulliford et al., 2006), it has a positive impact on the utilization of community medical service (Nutting et al., 2003). The continuity of medical care also helps patients maintain a better health and improve their medical satisfaction. A study by Lambrew et al. (1996) found that 91% of patients with poor health and a fixed source of medical care reported that they have been receiving a doctor’s consultation at least once a year. In contrast, only 75% of patients with poor health and no regular source of medical care said they have annual doctor visits. Therefore, contracting community family doctors has become an important starting point for optimizing the rational allocation of health resources, and has an important role in promoting hierarchical medical system and improving the coverage of primary treatment in community hospitals. Some studies have analyzed the willingness of Beijing residents to seek medical treatment in community hospitals. It found that the contracting of family doctors has a significant impact on residents’ willingness to seek medical treatment in community health institutions (Shi et al., 2016). At the same time, some studies have found that the residents who have signed contracts have become the core population of the primary treatment in community health institutions. The effectiveness of community family doctor contract directly affects the willingness of residents to go to the community hospital for the primary treatment (Juan et al., 2017).

Analyzing the medical consumption behavior of residents from the perspective of economics, the residents’ demand for medical services can be regarded as the induced needs caused by the “health” demand. People often determine their desired health stock level according to the price of medical services they need to bear (Grossman, 1972). The traditional medical choice model believes that medical services are ordinary goods, so when the proportion of medical insurance reimbursement is high and the price paid by patients is low, patients are more willing to consume medical services. The “Rand Experiment” and its follow-up studies have both verified this hypothesis that there is a significant negative correlation between residents’ medical demand and out-of-pocket expenses (Keeler and Rolph, 1988). With the continuous improvement of medical insurance coverage, the concept of patient cost sharing derived from medical service prices has attracted the attention of policymakers and scholars (Chandra et al., 2010). Some scholars conducted a study on supplementary insurance for the California public employees after retirement, and found that the increase in the deductible amount will significantly reduce the frequency of patients’ visits and medication costs (Shigeoka, 2014). An empirical study on outpatient and inpatient expenses of the elderly in Japan found that a decrease in the proportion of out-of-pocket payments would increase the consumption of medical resources. Domestic-related empirical studies have also found that the proportion of medical insurance reimbursement has a significant impact on the visits of residents in community hospitals (Xie et al., 2010; Zhong et al., 2016).

At the same time, there is heterogeneity in the preference of medical services for different patient characteristics. Except for the elderly who has a rigid demand for the continuity of daily health care and nursing due to the decline of physical function, non-elderly patients with chronic diseases also have significantly higher demand for family doctor contracts and primary medical and health resources than other groups due to their daily care needs “China’s Medium and Long-term Plan for the Prevention and Treatment of Chronic Diseases (2017–2025)” proposes that the community health service centers should focus on individualized health interventions for chronic diseases, and gradually realize its early screening and early intervention, and conduct health management for chronically ill patients; the Plan also proposes that patients with chronic diseases should be given priority to be included in the contracted services of family doctors, and encourages them to seek medical treatment in community health institutions. The continuity of medical care required by chronic diseases and the availability of medical resources also make these patients more inclined to community health institutions than patients with other diseases. Studies have shown that the contracting rate of chronic disease patients and their intention to seek medical treatment in community medical institutions are both higher than those of other community residents (Lu et al., 2016; Wu et al., 2021). On the one hand, due to the encouragement of relevant policies, family doctors are encouraged to sign contracts with chronic disease patients, who are viewed as key groups; on the other hand, due to their own health care needs, patients with chronic diseases have a relatively high willingness to sign the contracts (Xu et al., 2018). Therefore, patients with chronic diseases are more willing to enjoy family doctor services by signing contracts to improve their quality of life and daily living ability through health management, and are more inclined to visit doctors and obtain medicines regularly in community health service institutions.

Based on case studies, questionnaire surveys, and so on, the existing studies have carried out rich discussions on the expand of contracting family doctors in China and its impact on the medical treatment behavior of residents in the community. However, these studies mainly focus on the subjective feelings of the interviewed residents or their cognition of the family doctor contract, few studies use the actual medical data and detailed medical insurance settlement data for empirical analysis. For healthy young adults, even if they have signed a family doctor contract, and even if their willingness to respond to this service and their awareness of it are both higher, the impact of the contracting behavior on their visit frequency and medical expenses of community hospital may also be relatively limited. Therefore, resident contracting is only the first link of family doctor services and the hierarchical treatment of community hospitals. What kinds of factors interfere with residents’ medical treatment behavior after the contract is signed, and whether the family doctor contracting has an effect on key groups such as the elderly and chronic patients, still needs to be further discussed, in particular, requires empirical testing using the actual medical treatment record data and medical insurance settlement data of residents.

## Materials and methods

### Sample selection and data sources

As early as 2010, Beijing proposed to “strengthen the function of medical service of the primary medical and health institutions and promote the family doctor-style medical service”; with the continuous exploration of the service model of family doctor contract, the signing rate of Beijing community residents, especially the elderly, has been increasing for many years. According to the relevant data from the National Health Commission in 2021, more than 70% of the population over 65 in Beijing has completed the signing of family doctor services. In order to explore the impact and impact mechanism of family doctor contracted services on residents’ medical expenses, this paper uses the monthly data of 5,851 residents in a community hospital in Beijing for a total of 41 months from January 2018 to May 2021 for empirical testing. The sample data used in this paper involve four types of medical insurance: self-pay, local medical insurance, free medical service, and non-local medical insurance. The reimbursement ratio of each type is different. Due to the difference in medical expenses paid by the patient, it may cause the difference of patient’s “desirable” health stock and corresponding spending on health investment, resulting in different needs for medical services.

### Model building and variable description

In order to test the impact of community family doctor signing on residents’ medical expenses, the basic regression model is set as follows:


(1) $${\text{Fee}}_{i.t}=\beta_{0}+\beta_{1}\times {\text{sign}}_{i,t}+\beta_{2}\times {\text{control}}_{i,t}+\varepsilon_{i,t}$$

Among them, “fee” is the monthly medical expenses of each resident in the community hospital, “sign” is a binary variable (0 means not signed, 1 means signed), and at the same time, the basic characteristics of the residents (age, gender, whether with a chronic disease), the types of medical insurance, the times of visits per month, and the number of departments visited are set as control variables. Further, in order to explore the impact mechanism of community family doctor contracting on residents’ medical expenses, the interaction items of contracting and the times of visits and departments patients visited are added on the basis of model (1). Its theoretical mechanism is that family doctor service contract strengthens the connection between doctors and patients, and improves residents’ understanding of community health institutions, so that residents are more willing to choose community hospitals for diagnosis and treatment or daily health care; the data show that the monthly frequency of medical visits and the number of medical departments residents visited have increased. In model (2) and model (3), we mainly focus on the plus or minus of the coefficient of the interaction terms. If the coefficient is plus, it means that the contracting of family doctor services has improved the utilization of residents’ medical services in community hospitals. See Table 1 for a description of these relevant variables.


(2) $$\begin{array}{l} {\text{Fee}}_{i,t}=\beta_{0}+\beta_{1}\times \text{sign}*{\text{times}}_{i,t}+\\ \;\beta_{2}\times {\text{sign}}_{i,t}+\beta_{3}\times {\text{control}}_{i,t}+\varepsilon_{i,t}\; \end{array}$$

(3) $$\begin{array}{l} {\text{Fee}}_{i,t}=\beta_{0}+\beta_{1}\times \text{sign}*{\text{numbers}}_{i,t}+\\ \;\beta_{2}\times {\text{sign}}_{i,t}+\beta_{3}\times {\text{control}}_{i,t}+\varepsilon_{i,t}\; \end{array}$$

**Table 1:** Description of relevant variables.

Variables	Symbol	Variables description
Medical expenses	Fee	Total monthly medical expenses of each resident
Whether contract the community family doctor	Sign	Contracted is assigned the value of 1, not contracted is assigned the value of 0
Age	Age	Continuous variable
Gender	Male	Male is assigned the value of 1, female is assigned the value of 0
Types of medical insurance	Type	1 = non-local medical insurance, 2 = new rural cooperative medical care system, 3 = medical insurance for non-working urban residents 4 = medical insurance for working urban residents, 5 = free medical service (including retired cadres and residents with preferential treatments); 1–5 were assigned value according to reimbursement ratio from low to high
Whether with a chronic disease	Chronic	If the diagnosis result is a chronic disease, the value is 1, otherwise 0
The times of visits per month	Times	The total times of medical visits per resident per month
The number of departments visited	Numbers	The total number of departments visited by each resident per month

The results of descriptive statistics of the main variables in the sample data are shown in Table 2. The sample data in this article are patients with medical records in the community hospital and at least one visit per year during the observation period (2018–2021). There are 5,916 patients who meet the conditions for sample entry. A total of 238,456 pieces of monthly resident medical treatment data for all samples were collected during the observation period of 41 natural months. From Table 2, the basic information of the patients entering the samples can be observed. Judging from the information on the medical expenses of each patient, there is a big difference between the minimum value and the maximum value, and the variance is as high as 817.5, indicating that there is a great difference in the expenses per person per visit. Judging from the data of contracting, there are 3,686 residents who have signed contracts in the samples, and the signing rate is 63.4%. The proportion of male patients in the samples was 43.7%, and the proportion of male and female patients tend to be average. In terms of the distribution of age, the average age of the patients is (56.53 ± 19.09) years old. Chronic disease patients accounted for 42.9%. The medical insurance types of the sample residents include five: non-local medical insurance, new rural cooperative medical care system, medical insurance for urban non-working residents, medical insurance for urban working residents, and free medical service. From the perspective of variance, the distribution of medical insurance types is relatively balanced, and the proportion of extreme values such as non-local medical insurance and free medical service is relatively low. Overall, the difference in the proportion of residents’ medical insurance reimbursement is relatively small. From the perspective of residents’ utilization of community medical services, there are large differences in the times of residents’ visits per month, with the highest frequency of visits being 34 per month (The settlement system of the community hospital records the bills generated by different departments in the same medical visit as different number of visits. If the bill for the same visit involves 3 different departments, it will generate 3 medical visits records), the maximum number of medical departments visited per month is 5, and the variance is 0.648.

**Table 2:** The descriptive statistics of main variables.

Variables	Sample size	Mean	Variance	Minimum value	Maximum value
Fee	238,456	571.2	817.5	0	11,807
Sign	238,456	0.634	0.482	0	1
Male	238,456	0.437	0.496	0	1
Age	238,456	56.53	19.09	0	98
Chronic	238,169	0.429	0.495	0	1
Type	238,456	3.839	0.462	1	5
Times	238,456	1.943	2.571	0	34
Numbers	238,456	0.660	0.648	0	5

## Results

### The impact of community family doctor contract on medical expenses

Based on model (1) to describe the relationship between community family doctor contract and residents’ medical expenses, columns (1) and (2) in Table 3 show the impact of community family doctor contract on residents’ medical expenses, of which column (1) is the result of OLS regression, column (2) is the regression result of the random effects model controlling for the effect of time. From the data listed in the table, it can be seen that after controlling for the times of monthly visits and the number of medical departments visited, the contract of community family doctors is significantly related to the monthly medical consumption expenditure of residents. Specifically, the average monthly medical expenditure of contracted residents in the OLS regression increased by 61.97%, while the contracted residents in the random effects model increased by 121.55%. This shows that residents who have contracted a family doctor service are more willing to seek medical treatment in a community hospital, or that the family doctor contract significantly improves the service availability of the community hospital to the community residents and effectively improves the utilization of the community hospital’s medical services, which is conducive to promoting the coverage of the hierarchical medical system. This is consistent with the empirical results obtained by Shi et al. (2016) based on a questionnaire survey of residents in Xicheng District, Beijing: the contracting of community family doctors has a significant impact on residents’ willingness to seek medical treatment in community hospitals. In terms of control variables, age, the times of visits per month, and the number of departments residents visited per month are all significantly positively correlated with medical expenses, that is, the elderly residents pay more for medical expenses in community hospitals. The more the times of visits and the more the number of departments visited, the higher the monthly medical expenses. In addition, the types of medical insurance have a significant positive correlation with medical expenses. This result is consistent with the empirical research result of Sun et al. (2020), that is residents’ medical treatment behavior is affected by different types of medical insurance.

**Table 3:** The regression result of model (1).

Variables	OLS	RE
Sign	29.963*** (13.94)	70.963*** (9.76)
Male	6.798*** (3.57)	−2.697 (−0.39)
Age	0.575*** (10.66)	0.620*** (3.14)
Chronic	80.678*** (35.84)	88.886*** (10.98)
Type	46.152*** (24.26)	70.372*** (10.61)
Times	228.121*** (191.44)	198.066*** (116.88)
Numbers	123.521*** (35.66)	114.498*** (24.42)
Constant	−454.481*** (−43.78)	−473.099*** (−19.12)
Observations	238,169	238,169
*R*-squared	0.671	

*** indicate significant at 1%, respectively, and the values in () are the standard errors of the regression coefficients.

In order to test the applicability of the random effect model to the data in this paper, we further performed the LM test on the mixed OLS model and the random effect model. The value of *P* is 0, indicating that the model has random effects. Therefore, the random effect model is selected for the estimated parameters below. At the same time, this paper considers the effect of time on the basis of the random effect model.

### Impact mechanism of community family doctor contract on medical expenses

The previous results of benchmark regression show that the family doctor service contract significantly increases the medical expenses of residents in community hospitals, so how does the contracting behavior affect the medical expenses? On the one hand, one possible mechanism is that residents have formed a more stable doctor-patient relationship with family doctors after signing the contract, so they are more inclined to choose the hospital where the family doctor is working at when needing a medical treatment. On the other hand, through the community family doctor service, the residents’ awareness of the related services of the community hospital has been strengthened, and the service utilization of the residents in the community hospital has also been improved. Therefore, on the basis of model (1), we add the interaction items of whether to sign a contract and the times of visits and the number of departments patients visited in this section, to test the impact mechanism of contract on medical expenses.

Columns (2) and (3) in Table 4 are the results of adding the interaction items sign*times and sign*number, respectively. The interaction items in both models are significantly positive, which indicates that the frequency of residents’ visits and the number of departments visited to community hospitals after signing the contract increase significantly. This is consistent with the findings of existing studies that the number of doctor visits of contracted residents is significantly higher than that of non-contracted residents (Chen et al., 2017). At the same time, the results of columns (2) and (3) show that basic residents’ indicators such as age, whether patients with chronic diseases, and the types of medical insurance are still statistically significant at the confidence level of 1% after adding interaction items, indicating that the two factors of physical condition of residents, age and chronic diseases, as well as the proportion of medical insurance reimbursement, still have significant effects on medical expenses.

**Table 4:** The regression results after adding the interaction items.

Variables	(1)	(2)	(3)
Sign*number			58.059*** (6.04)
Sign*times		24.525*** (6.95)	
Sign	70.963*** (9.76)	34.925*** (5.28)	39.695*** (5.98)
Male	−2.697 (−0.39)	−2.006 (−0.29)	−2.307 (−0.33)
Age	0.620*** (3.14)	0.576*** (2.95)	0.583*** (2.97)
Chronic	88.886*** (10.98)	91.299*** (11.31)	90.957*** (11.26)
Type	70.372*** (10.61)	81.888*** (12.42)	80.519*** (12.22)
Times	198.066*** (116.88)	177.006*** (52.19)	197.978*** (116.93)
Numbers	114.498*** (24.42)	120.782*** (25.81)	71.069*** (8.08)
Constant	−473.099*** (−19.12)	−484.273*** (−19.57)	−484.526*** (−19.55)
Observations	238,169	238,169	238,169

*, *** indicate significant at 10, and 1%, respectively, and the values in () are the standard errors of the regression coefficients.

### Heterogeneity analysis

The above analysis focuses on the factors that affect the medical expenses of community hospitals in the whole samples of residents. The basic conclusions are: first, the contract has a significant impact on the medical expenses of residents in the community hospital; further, signing the contract has an impact on residents’ medical expenses in community hospitals by increasing the frequency of residents’ visits in community hospitals and the number of departments they visit. At the same time, the regression results show that the two control variables of age and whether patients with a chronic disease have a statistically significant positive impact on medical expenses. Therefore, in the part of heterogeneity analysis, we will focus on exploring the differences between the influencing factors of the medical expenses of the elderly and patients with chronic diseases among the contracted residents and the other residents.

Table 5 lists the regression results of all contracted residents samples using model (1), as well as grouped by age (≥65 is the group of elderly) and whether patients with chronic diseases.

**Table 5:** Regression results of contracted samples grouped by age and chronic disease.

Variables	(1)	(2)	(3)	(4)	(5)
	All	The old	Others	Chronic	Others
Male	−3.475 (−0.35)	2.821 (0.19)	−8.836 (−0.65)	−0.686 (−0.05)	−4.291 (−0.31)
Age	0.829*** (3.52)			0.572* (1.76)	1.093*** (3.22)
Chronic	86.184*** (8.54)	72.461*** (5.04)	97.957*** (6.89)		
Type	88.608*** (6.53)	69.995*** (3.54)	113.022*** (6.03)	38.983** (2.06)	126.057*** (6.54)
Times	201.578*** (109.56)	207.688*** (84.44)	194.503*** (71.66)	203.988*** (86.71)	200.181*** (69.70)
Numbers	118.419*** (22.45)	122.252*** (17.16)	116.795*** (14.92)	139.700*** (19.82)	97.826*** (12.43)
Constant	−428.612*** (−7.94)	−285.905*** (−3.54)	−495.504*** (−6.83)	−56.780 (−0.71)	−676.879*** (−9.05)
Observations	151,044	79,089	71,955	79,704	71,422

*, **, *** indicate significant at 10, 5, and 1%, respectively, and the values in () are the standard errors of the regression coefficients.

The comparison of the coefficients of column (2) with column (1) and column (3) shows that the times of medical visits of the elderly among the contracted residents is significantly higher than that of other age groups, which indicates that for the elderly group with greater demand for medical resources, they are more willing to choose the nearest community hospital for treatment if they have signed a contract. The regression results are consistent with the stepwise regression results of Huang et al. (2019). It shows that the contracted service of family doctors has significantly improved the utilization of medical services for the elderly population in their region; at the same time, the elderly choosing more community hospitals for medical treatment is also an important part of promoting the hierarchical medical system and optimizing the allocation of medical services. The empirical evidence in Table 5 once again verifies that the contracted service of family doctors can be used as the key to promote the hierarchical treatment. In addition, the regression results in column (2) show that the coefficient of insurance types for elderly residents who have signed contracts is significantly positive, indicating that the medical expenses of the elderly population in China are significantly related to the proportion of medical insurance reimbursement, which is consistent with the empirical conclusions of existing research: the medical expenses of the elderly in China are significantly affected by the proportion of out-of-pocket payments, or the medical expenses of the elderly are significantly related to the proportion of medical insurance reimbursement (Liu and Qiu, 2014). At the same time, the comparison of coefficients in column (2), column (1), and column (3) shows that for the elderly, the types of medical insurance and whether they have chronic diseases have less impact on medical expenses than all contracted groups and other age of residents. Its impact mechanism is that with the increase of age, the decline of the elderly’s physical function brings a strong demand for daily health care, and the incidence of chronic diseases such as hypertension and coronary heart disease in the elderly population is higher than that of other age groups. Therefore, on the one hand, for the elderly, regardless of the reimbursement ratio, there is still a need to choose the nearest community hospital for daily health care; on the other hand, the impact of chronic disease on medical costs varies less in the older population than in other age groups. The comparison of the coefficients in column (4) with those in columns (1) and (5) shows that the times of clinic visits and the number of departments they visited in community hospitals for contracted chronic disease patients are significantly higher than all contracted samples and non-chronic disease patients. This shows that, as the key care object of family doctor contracted services, signing has indeed significantly improved the utilization of community medical services for patients with chronic diseases; it provides data support for the current family doctor contracted services to carry out promotion work among residents with chronic disease as important key groups. Similar to the regression results of the elderly group, the significance and coefficient of medical insurance types in the chronic disease samples are both lower than those of all contracted samples and non-chronic disease patients, indicating that for chronic disease groups with long-term medical care needs, the level of out-of-pocket ratio has less impact on their expenses in community hospitals, and the continuity of care brought by the contracted services of family doctors and the accessibility of medical services provided by community hospitals are considered higher priority than the proportion of medical insurance reimbursement when the chronic disease group pays medical expenses. In addition, the factor of age in the chronic disease group also shows that the coefficient and significance were lower than all contracted samples and non-chronic disease groups. The theoretical mechanism is that regardless of whether people with chronic diseases are in the aging period, there is always a long-term demand for medical and health services due to their diseases, and the influence of age on medical expenses is greatly weakened in the chronic disease group. That is, chronic patients have long-term care and medical needs regardless of their advanced age.

### Robustness check

In this paper, the method of substituting variables is used to test the robustness of the aforementioned research conclusions. The robustness of the basic regression results is tested by substituting medical expenses (fee) and frequency of visits (times). The resident’s monthly medical expenses are replaced by the average value of the resident’s medical expenses in each quarter, and the monthly frequency of medical visits are substituted by the average times of residents’ visits in each quarter.

Table 6 reports the results of two kinds of regression, OLS, and random effects, based on model (1) after alternating variables. The results listed in the table show that after displacing the two main variables at the same time, controlling for the basic characteristics of residents (age, gender, whether or not patients with chronic diseases) and the types of medical insurance, the contracted services of community family doctors are still significantly positively correlated with residents’ medical expenses. That is, the main regression results have not changed substantially, and the aforementioned research conclusions are still robust. In addition, the results of the LM test after substituting key variables still indicate that the random effects model is more suitable for the interpretation of sample data. Table 7 reports the results of the heterogeneity test after replacing the dependent variable (fee) and the main control variable (times). The comparison of the coefficients in column (2) with those in columns (1) and (3) shows that after replacing the key indicators, the times of medical visits of the contracted elderly group is still significantly higher than that of all contracted residents and other age groups. The comparison of the coefficients in column (4), column (1), and column (5) shows that after the key indicators are replaced, the times of visits by contracted chronic disease patients is still significantly more than that of all contracted residents and non-chronic disease patients. That is, the results of the heterogeneity test that the contracting of family doctor services significantly improved the utilization of community health services for the elderly and chronic patients are still significant.

**Table 6:** Regression results of model (1) after substituting variables.

Variables	OLS	RE
Sign	4.693** (2.55)	61.537*** (8.01)
Male	17.691*** (11.28)	3.961 (0.57)
Age	0.575*** (12.80)	0.636*** (3.18)
Chronic	77.859*** (41.57)	88.992*** (10.87)
Type	35.325*** (22.20)	68.518*** (9.97)
Average times	251.275*** (253.27)	209.520*** (90.50)
Numbers	75.756*** (32.40)	68.795*** (26.27)
Constant	−271.199*** (−36.85)	−334.751*** (−13.44)
Observations	238,169	238,169
*R*-squared	0.703	

**, *** indicate significant at 5, and 1%, respectively, and the values in () are the standard errors of the regression coefficients.

**Table 7:** Grouping regression results after substituting variables.

Variables	(1)	(2)	(3)	(4)	(5)
	All	The old	Others	Chronic	Others
Male	5.526 (0.54)	12.352 (0.83)	−0.200 (−0.01)	9.161 (0.64)	3.940 (0.28)
Age	0.866*** (3.59)			0.617* (1.87)	1.093*** (3.11)
Chronic	88.075*** (8.57)	74.095*** (5.03)	100.589*** (7.02)		
Type	88.924*** (6.22)	72.355*** (3.44)	107.061*** (5.53)	35.410* (1.78)	124.873*** (6.21)
Average times	210.307*** (81.41)	214.657*** (61.55)	205.401*** (53.92)	211.403*** (58.81)	209.881*** (56.74)
Numbers	68.751*** (22.74)	67.888*** (16.65)	70.172*** (15.56)	75.514*** (18.12)	61.701*** (14.16)
Constant	−347.753*** (−6.14)	−215.624** (−2.52)	−382.336*** (−5.17)	−0.438 (−0.01)	−539.713*** (−6.93)
Observations	151,044	79,089	71,955	79,704	71,422

*, **, *** indicate significant at 10, 5, and 1%, respectively, and the values in () are the standard errors of the regression coefficients.

## Discussion and conclusion

With the vigorous promotion of community family doctor contract service in Beijing, the impact of it on medical expenses has been increasingly concerned by the society. This paper uses actual medical treatment records and monthly data of medical insurance settlement of a total of 5,851 residents in a community in Beijing from January 2018 to May 2021 to analyze the impact and impact mechanism of community family doctor contract on residents’ medical expenses. The study finds that the family doctor service contract significantly increased the monthly frequency of residents’ medical visits in community hospitals, especially for patients with chronic diseases and the elderly. It shows that it is scientific to a certain extent that the policy orientation of family doctor contract service promotion views the elderly and chronic disease patients as the main audience. At the same time, the data used in this paper are composed of the monthly medical insurance settlement details of the community hospital and the actual diagnosis and treatment records, which can match the actual medical expenses data and patient characteristic data one by one, so as to demonstrate the relationship between the contract of family doctors and the medical expenses of residents through empirical analysis, which has practical reference value.

Different from the existing studies which used questionnaire and other forms to obtain patients’ self-administered data, the data in this paper are based on the actual settlement records of the hospital system, which can avoid the bias caused by the data collection method to a certain extent, and provide more reliable empirical evidence to support the family doctor contracting system. However, this study also has some limitations. The study is based on monthly medical insurance settlement details of the community hospital and the actual diagnosis and treatment records, due to data availability issues and Privacy protection of patients, more demographic information could not be considered in the analyzes above. We would supplement relevant information and further investigate the impact of patient demographic characteristics on the effectiveness of the family physician contracting system in the future.

Meanwhile, the regression results are still robust after substituting key variables. Therefore, we can draw the following policy implications:

First, the contracted service of family doctors has a significant positive effect on improving the daily diagnosis and treatment of community residents in primary health service institutions. At present, the various forms of family doctor contracting services carried out in various places have positive significance for the formation of a reasonable medical order featuring primary treatment at the community level, flexible inter-hospital patient transfer, differentiated treatment for acute and chronic illnesses, and coordination across different levels.

Second, the reimbursement ratio of medical insurance plays a significant role in regulating the patient’s behavior of medical visits. Further promoting the rational optimization of medical resources can take the increasing of the medical insurance reimbursement ratio of primary health institutions and secondary and tertiary hospitals as an important starting point. Differences in reimbursement ratios will cause changes in residents’ preference for choosing different hospital grades when seeking medical treatment (Huang et al., 2019). At present, Beijing aims to implement the reform of separation of medical treatment and drug sales, and strives to increase the difference in the reimbursement ratio of medical insurance between community health service institutions and the secondary and tertiary hospitals, so as to strengthen the low-cost advantage of community hospitals. But overall, the cost difference is still small, and the relative advantages of community health institutions for patients are still slightly insufficient. At the same time, based on the “price-quality” model and hospital selection theory, the community health institutions should focus on solving quality problems such as lack of varieties of drugs, shortage of general practitioners, and insufficient of medical equipment, so as to eliminate patients’ concerns about the quality of diagnosis and treatment in community hospitals (Hu et al., 2020).

Third, we should focus on special groups such as the elderly and patients with chronic diseases, and these groups with higher needs for access to medical services and continuity of care should be the core subjects of family doctor contracted services, so that the primary medical resources will benefit more residents who need timely medical treatment, and the allocation of medical resources will also be more reasonable.

In conclusion, the family doctor contract service carried out in Beijing has played a positive role in improving the utilization of medical resources in community health institutions, and provided empirical evidence for further promoting the hierarchical medical system with the family doctor contract service as its starting point. It also provides an important reference for improving the scientificity and practicability of related policies and systems such as the family doctor contract and hierarchical medical system.

The advancement of the “first diagnosis in primary care followed by graded diagnosis and treatment” mechanism has long been a hot topic in China. As an essential policy, whether family doctor contracting services could effectively improve hierarchical medical system should be tested. Due to data availability issues, existing literatures has not provided sufficient empirical evidence to prove the effectiveness of family doctors.

There are lots of essential medical relevant features interfere with family doctors expect expenses. At present, domestic researches basically focus on the “gatekeeping” responsibility of family doctors in medical expenses. What kinds of medical-related factors affected by family doctors still need to be further analyzed. Meanwhile, whether demographic information of residences could impact the mechanism of family doctor contracting services and the extent of impact should be further discussed.

## Data availability statement

The raw data supporting the conclusions of this article will be made available by the authors, without undue reservation.

## Ethics statement

Ethical review and approval was not required for the study on human participants in accordance with the local legislation and institutional requirements.

## Author contributions

LL and XH carried out the study, analyzed the data, and drafted the manuscript. XH and LL were responsible for writing the literary and revising the language. CZ provided the guidance for revising the manuscript. All authors read and approved the final manuscript.

## Funding

This research is supported by National Natural Science Foundation of China (grant number: 72204251) and China Postdoctoral Science Foundation (grant number: 2022M713443). The funders had no role in study design, data collection, and analysis, decision to publish, or preparation of the manuscript.

## Conflict of interest

The authors declare that the research was conducted in the absence of any commercial or financial relationships that could be construed as a potential conflict of interest.

## Publisher’s note

All claims expressed in this article are solely those of the authors and do not necessarily represent those of their affiliated organizations, or those of the publisher, the editors and the reviewers. Any product that may be evaluated in this article, or claim that may be made by its manufacturer, is not guaranteed or endorsed by the publisher.
